# Spike-based Decision Learning of Nash Equilibria in Two-Player Games

**DOI:** 10.1371/journal.pcbi.1002691

**Published:** 2012-09-27

**Authors:** Johannes Friedrich, Walter Senn

**Affiliations:** Department of Physiology and Center for Cognition, Learning and Memory, University of Bern, Switzerland; Indiana University, United States of America

## Abstract

Humans and animals face decision tasks in an uncertain multi-agent environment where an agent's strategy may change in time due to the co-adaptation of others strategies. The neuronal substrate and the computational algorithms underlying such adaptive decision making, however, is largely unknown. We propose a population coding model of spiking neurons with a policy gradient procedure that successfully acquires optimal strategies for classical game-theoretical tasks. The suggested population reinforcement learning reproduces data from human behavioral experiments for the blackjack and the inspector game. It performs optimally according to a pure (deterministic) and mixed (stochastic) Nash equilibrium, respectively. In contrast, temporal-difference(TD)-learning, covariance-learning, and basic reinforcement learning fail to perform optimally for the stochastic strategy. Spike-based population reinforcement learning, shown to follow the stochastic reward gradient, is therefore a viable candidate to explain automated decision learning of a Nash equilibrium in two-player games.

## Author Summary

Socio-economic interactions are captured in a game theoretic framework by multiple agents acting on a pool of goods to maximize their own reward. Neuroeconomics tries to explain the agent's behavior in neuronal terms. Classical models in neuroeconomics use temporal-difference(TD)-learning. This algorithm incrementally updates values of state-action pairs, and actions are selected according to a value-based policy. In contrast, policy gradient methods do not introduce values as intermediate steps, but directly derive an action selection policy which maximizes the total expected reward. We consider a decision making network consisting of a population of neurons which, upon presentation of a spatio-temporal spike pattern, encodes binary actions by the population output spike trains and a subsequent majority vote. The action selection policy is parametrized by the strengths of synapses projecting to the population neurons. A gradient learning rule is derived which modifies these synaptic strengths and which depends on four factors, the pre- and postsynaptic activities, the action and the reward. We show that for classical game-theoretical tasks our decision making network endowed with the four-factor learning rule leads to Nash-optimal action selections. It also mimics human decision learning for these same tasks.

## Introduction

Neuroeconomics is an interdisciplinary research field that tries to explain human decision making in neuronal terms. Behavioral outcomes are construed as results of brain activity and the neuronal correlates of the quantities relevant for the decision making process are identified. Humans, as economic agents, attempt to optimize some reward function by participating in the production, exchange and maintenance of goods. Reward for the individuals will depend in general not merely upon their own actions but also on those of the other players and, furthermore, these will adapt their own strategies.

Classical models in neuroeconomics are based on temporal difference (TD) learning [1], an algorithm to maximize the total expected reward [2] with potential neuronal implementations [3], [4]. It assumes that the environment can be described as a Markov decision process (MDP), i.e. by a finite number of states with fixed transition probabilities [5]. Multi-agent games, however, are not Markovian as the evolution of the environment typically does not only depend on the current state, but also on the history and on the adaptation of the other agents. Such games can be described as partially observable Markov decision processes (POMDP, [6]) by embedding the sequences and the learning strategies of the other agents into a large state space. We have presented a policy gradient method for population reinforcement learning which, unlike TD-learning, can cope with POMDPs and can be implemented in neuronal terms [7]. Yet, since a human learner would need to successfully explore the large state space of the POMDP, this appears to be an unrealistic scenario for explaining decision making in a multi-agent environment. A more realistic learning scenario is that humans transiently conceive the other players to follow a fixed strategy, and try to find their optimal counter strategy under this stationarity approximation. Maximizing one's own payoff while assuming stationarity in the opponents strategy is called a fictitious play and conditions are studied when this play effectively converges to a stationary (Nash) equilibrium [8].

Here we show that for classical two-player games [9] a simplified population reinforcement learning approach [7], which is policy gradient under the stationarity approximation, can reproduce human data. We consider two games, blackjack [10] and the inspector game [11], as examples for which the optimal strategy is either deterministic or stochastic, respectively. Optimality is expressed in terms of the Nash equilibrium, a solution concept for games involving two or more players. It is reached when no player has anything to gain by changing its strategy unilaterally. Each player is making the best decision it can, taking into account the decisions of the other(s), hence the Nash equilibrium constitutes an optimum. Our algorithm is consistent with behavioral experiments for these games [10], [11] while performing optimally according to the Nash equilibrium. We also show that TD-learning as well as covariance learning fail to find the stochastic Nash equilibrium for the inspector game.

The current paper follows a long tradition of explaining human and animal behavior by simple models of reward-based learning, starting from Thorndike's law of effect [12] and Pavlovian conditioning paradigms [13], [14] up to more recent theories of reinforcement learning [1], [2], [15], [16]. Basic reinforcement learning with simple models of a few free parameters have also been applied to games. It has been shown for a wide set of two-player games that these simple algorithms well approximate human performance [17].Yet, we show that basic reinforcement learning does not follow the reward gradient, and in fact it does not fit human data on the inspector game as well as our gradient rule. Obviously, playing games involves cognitive reasoning, as for instance captured by the theory of ‘adaptive control of thought–rational’ (ACT-R, [18]). Within such a theory, our model represents a neuronal implementation of a ‘production rule’ which initiates a behavioral pattern in response to sensory and possibly cognitive input.

## Results

### Model

**Figure 1:**
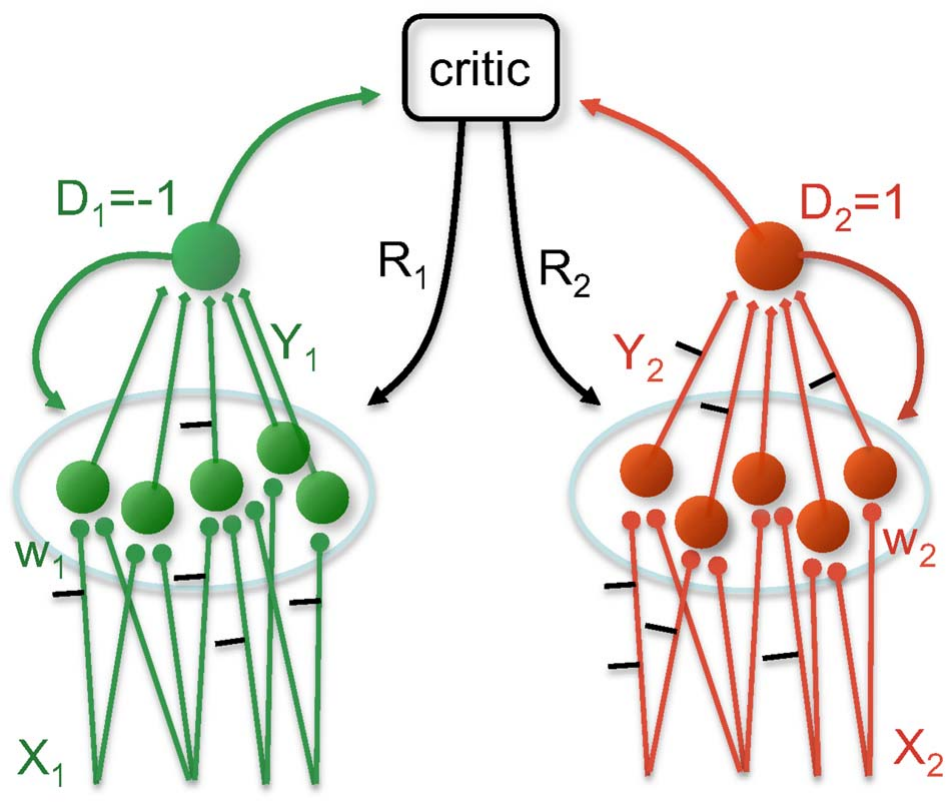
Neuronal architecture implementing the two players. Each player 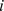 (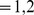) is represented by a population of decision making neurons (shown 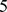 of each) which receive an input spike pattern 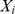 and generate an output spike pattern 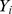. The population decision 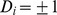 is represented by a readout unit, with 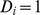 being more likely when more decision making neurons fire at least one output spike. The synaptic weights 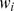 are adapted as a function of 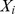, 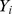, 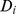 and the reward signal 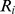 delivered by a critic.

The network representing a player consists of a population of 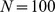 Spike-Response-Model (SRM) neurons with escape noise [19], driven by a common presynaptic stimulus encoding the current state of the game (the player's hand value in blackjack, and a fixed stimulus for the inspector game). Each input spike pattern (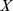) is composed of 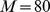 afferent spike trains generated once by independent 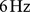 Poisson processes with 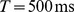 duration, and then repeatedly presented with the same fixed spike timings. The population neurons integrate the afferent presynaptic input spike trains and produce an output spike pattern (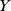, see Fig. 1). The decision of an individual postsynaptic neuron is denoted by 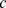, with 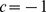, if the considered neuron does not spike, otherwise 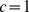. Behavioral decisions 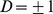 are stochastically made based on the population activity 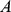 defined as sum of the individual decisions 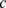 across the 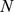 population neurons: if 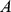 is small, the population decision is likely 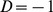, and the larger 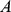 is, the more likely is 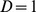. At the end of a game involving either a single decision (like in the inspector game) or a sequence of decisions (like in blackjack), a reward signal 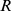 is delivered by an external critic which either informs about winning or losing (with 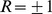 like in blackjack) or delivers a specific payoff (like in the inspector game).

The synapses feeding the stimulating spike pattern to the population neurons are updated according to a multi-factor plasticity rule involving the reward, the behavioral decision, the single neuron decision and the eligibility trace which depends on the post- and pre-synaptic activity:
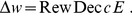
(1)Here, 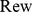 is the reward signal encoding the reward prediction error [20] (see Eq. 5 in Methods), 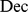 is the global feedback signal informing the synapses about the population decision 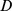 weighted by the population activity (Eq. 6 in Methods), and 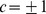 is the neuronal decision (spike/no spike). The eligibility trace 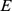 is a synaptic buffer roughly encoding the covariance between the past pre- and postsynaptic activity relevant for learning (Eq. 8 in Methods). Technically, 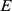 is the derivative of the log-likelihood of producing the postsynaptic spike train. The learning rule can be shown to perform gradient ascent in the expected reward (Supporting Text S2).

While most of the terms in Eq. 1 may have their standard biological counterpart [16], there is less experimental evidence for assigning the decision feedback 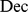
' to one specific neuromodulator. Yet, be the population neurons recurrently connected [21] or not, decision learning based on a population always requires that a global population signal is fed back to the individual neurons, as otherwise learning would quickly degrade with increasing population size [7], [22]. By the same performance reasons it is not possible to replace the other factors ‘
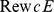
’ in Eq. 1 by a classical spike-timing dependent plasticity (STDP) implementation endowed with the multiplicative reward signal ‘
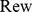
’ [23]. In fact, reward-modulated STDP is only able to learn multiple stimulus-response associations when the reward factor averages out to zero for each stimulus individually, requiring an additional reward-prediction network [16].

Our neuronal implementation is as simple as possible to provide the required computational properties. The lack of feedback connectivity avoids issues relating to population spike correlations [24], and the neural mechanisms supporting the readout of the decision and the population feedback signal are not considered here. Similarly, the fixed spike trains representing an input pattern is a biological simplification which does not fundamentally restrict the suggested approach.

### Blackjack

The simplified version of blackjack considered here was played in 18th century France and is the precursor of the version played in casinos nowadays. The card decks used consist of 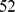 cards. Ace counts eleven, jack, queen and king ten points and the numbers two to ten according to their written value. The player (gambler) draws one card after the other, starting with an initial two card hand, with the object of bringing the hand value (total across drawn cards) as close as possible to 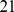, but stopping early enough so that it does not exceed this number, in which case he immediately loses. Afterwards the croupier does the same for the bank. The player wins if its score is higher than that of the croupier or if the croupier exceeds 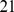, otherwise the croupier wins. The winner's payoff is 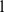, the loser's 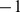. We assume that both player and croupier base their decision whether to draw another card or not only on their current hand value. Player and bank follow a strategy defined by the hand value 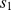 and 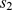, respectively, from which on they stop to draw another card.

The described rules of the game result in the payoff-matrix (Table 1) comprising the average payoff of the bank as a function of the strategies 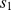 and 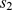 of the player and bank, respectively (Methods). The gambler loses whatever the bank wins, therefore the game is an example of a zero sum game. For zero sum games a Nash equilibrium corresponds to a minimax solution [25]. If the pay-off matrix has a saddle point (an entry which is the maximum in its row and the minimum in its column) the corresponding strategy pair is a minimax solution which represents a pure Nash equilibrium. In blackjack there is a unique such pair, 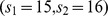, and hence there is a unique Nash equilibrium at all. For this optimal strategy pair the gambler stops drawing another card as soon as he has 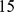 points or more, while the croupier stops at 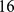 or more. The entry represents the lowest loss for the gambler given the strategy of the bank (minimum in the column), and the maximal payoff obtainable by the bank given the strategy of the gambler (maximum in the row). The Nash equilibrium is asymmetric because in the case of a standoff (equal final hand values) the croupier always obtains reward 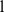 and the player 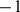. For hand values smaller than 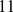 it is safe to draw another card whereas for more than 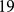 drawing another card leads to certain loss due to exceeding 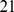. While we do not model these trivial actions, we address the learning problem for hand values between 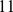 and 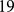.

**Table 1:** Average bank payoff for our version of blackjack.

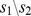	13	14	15	*16*	17	18	19
11	0.2982	0.3164	0.3027	*0.2544*	0.1689	0.0436	−0.1237
12	0.1635	0.2015	0.2076	*0.1791*	0.1130	0.0066	−0.1427
13	0.1052	0.1587	0.1806	*0.1679*	0.1176	0.0266	−0.1077
14	0.0438	0.1134	0.1536	*0.1597*	0.1282	0.0560	−0.0598
*15*	*0.0119*	*0.0706*	*0.1289*	**0.1555**	*0.1450*	*0.0940*	*−0.0008*
16	0.0143	0.0607	0.1085	*0.1557*	0.1685	0.1411	0.0702
17	0.0543	0.0893	0.1254	*0.1628*	0.1989	0.1980	0.1539
18	0.1349	0.1598	0.1854	*0.2120*	0.2394	0.2651	0.2509

The values show the calculated mean gains of the bank, respectively losses of the gambler, dependent on the strategy of the gambler (stopping to draw a card at hand values equal or larger than 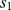) and the croupier (stopping at 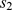). The strategies are described by the hand values from which on the players stop to draw another card. Formatted typesetting is used to highlight the Nash equilibrium.

#### pRL and TD-learning converge to a pure Nash equilibrium

**Figure 2:**
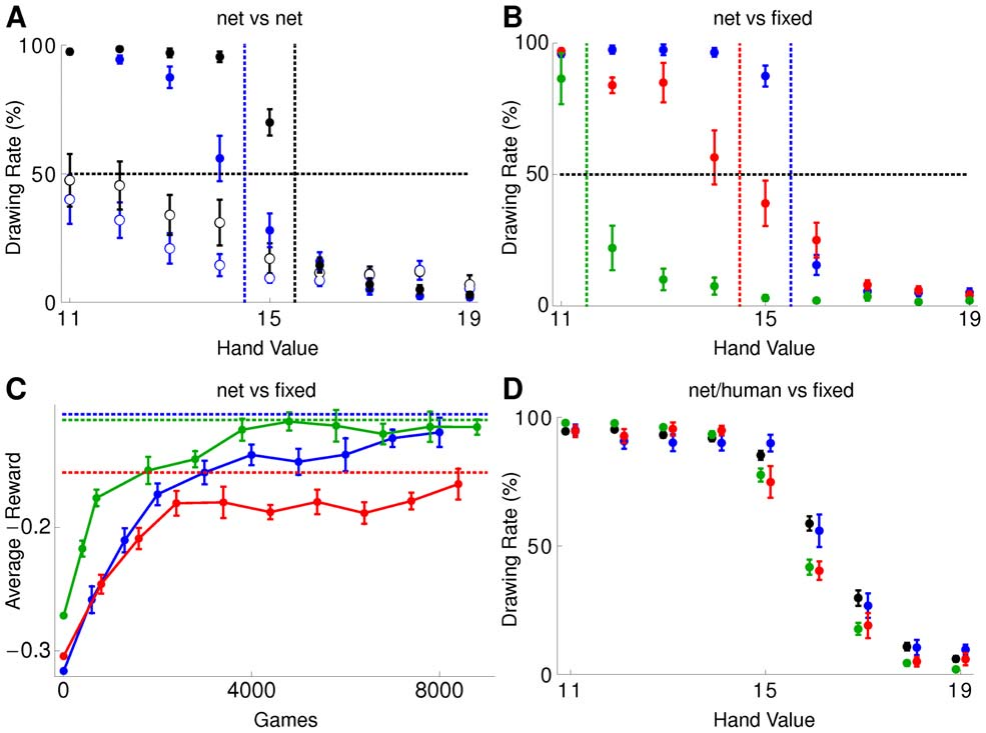
Playing blackjack with pRL converges toward pure Nash equilibrium. (A) Average strategy (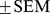) after 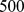 (open circles) and 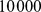 (filled circles) games where the gambler (blue) is a neural net as well as the croupier (black). The dotted vertical lines left of 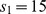 and 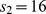 show the separation line of drawing/not drawing another card for the optimal Nash strategy pair. (B) Average strategy (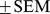) after 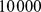 games for a neural net as gambler playing against a croupier that follows a given strategy 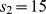 (blue), 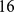 (red) or 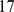 (green). The colored dotted lines left of 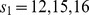 show the separation line of drawing/not drawing another card for the optimal strategy given that the croupier stops drawing at 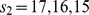 (from left to right). (C) Average reward (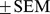) of the gambler for the scenario described in (B). The colored dotted lines show the maximal reachable average reward. (D) Average strategy (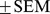) over the last 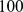 out of a total of 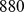 games for a neural net (red) or human (green) as gambler playing against a croupier that follows a given strategy 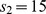. The initial weights of the network were chosen such that the strategy in the first 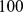 trials (blue) mimics the strategy of humans instructed about the game rules (black).

We first simulated two neural networks playing against each other. Each hand value between 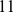 and 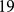 was represented by a fixed spatio-temporal spike pattern generated by 6 Hz Poisson processes, with different (but fixed) patterns distinguishing numbers, gambler and croupier. Since initially no ordering information is associated to the Poisson spike train encoding of the hand values, the learning process has yet to assign this information by trial and error. The drawing probabilities for each hand value learned by the gambler after 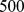 and 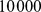 games, averaged over ten runs, are shown in Fig. 2A. The colored dashed lines in the plot indicate the decision boundaries of Nash equilibrium above which no further card is drawn by the gambler or croupier, respectively. Initially, both players randomly decide with 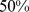 chance to draw (black dashed line). After about 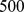 games both players have learned to not exceed 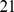 and do not draw further cards for high hand values, being still undetermined about what action to take for low hand values. After 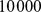 games both have learned successfully to draw another card for low hand values and tend to play according to the Nash equilibrium.

We next simulated the gambler by a neural net and the croupier by a computer algorithm which follows right from the beginning a fixed strategy. The resulting drawing probabilities after 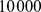 games are shown in Fig. 2B for three different strategies of the croupier, 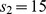, 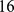 and 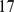. The gambler learns the perfect response strategies. The neural net exploits deviations of the croupier from the Nash equilibrium 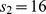 by also deviating and thus increasing its reward. If the croupier stops earlier at 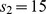 the gambler continues until 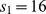, trying to exceed the croupier's hand value (blue), whereas if the croupier stops later at 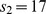 the gambler stops already at 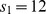 (green), taking advantage of the fact that the croupier likely exceeds 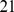. For the case 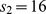 (red), the gambler does not learn the optimal strategy as well as for the two others. This is due to the fact that here the true mean rewards for the strategies 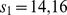 are close to the one for the optimal 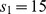, cf. Table 1, and cannot be distinguished based on merely 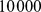 samples. Instead of just looking at the strategy we hence consider the maximally possible and the actually obtained reward for the three croupier strategies. Fig. 2C depicts the low pass filtered reward which approaches the theoretical optimum indicated by the dashed lines and read out from the corresponding columns 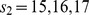 in Table 1. For the non-optimal croupier strategies (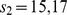) the maximally possible reward is significantly higher.

Hence, from playing against another network and against the croupier, we conclude that a neuronal population endowed with the plasticity rule (1) is able to learn the optimal strategies in blackjack, determined either by the pure Nash equilibrium, or by the croupier's fixed strategy. Replacement of the neural net by a TD-learner yields similar results for both scenarios (Supporting Text S1).

#### pRL fits human data on blackjack

Human behavior in blackjack was studied in [10], [26], though with a deck of 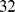 cards. The instruction of the subjects about the rules of the game already induces a prior in the drawing behavior with a preference for drawing or stopping in the case of low or high hand values, respectively. Neither pRL nor TD can reproduce this type of learning by insight. Moreover, the network needs first to learn the ordering of the stimuli by trial-and-error, and hence much more learning trials are required for the network. To still allow for a comparison with the human data, we used the same deck of 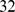 cards and simulated a neural network with initial weights chosen in such a way, that the initial strategy mimics the one of human's (Fig. 2D). After playing the same number of games as humans did, the network's final strategy agrees with the experimental data of humans, showing the same shift to a slightly less risky drawing behavior.

### Inspector game

The inspector game [27] has been widely studied in neuroeconomics [28]. The economic story surrounding the game is that a lazy employee prefers not to work. An employer knows this and sometimes ‘inspects’, but has to pay some cost ‘
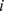
’for inspection. The payoffs for employee and employer are shown in Table 2. The inspector game shows only a mixed Nash equilibrium in which decisions are taken stochastically with a fixed probability. At the equilibrium, the players mix pure strategies, each with the same payoff: had these pure strategies different payoffs, then it would be better to just follow the pure strategy with the highest expected payoff.

**Table 2:** Payoff matrix of the inspector game.

employee\ *employer*	*inspect*	*Don't inspect*
work	0.5, *2-i*	0.5, *2*
shirk	0, *1-i*	1, *0*

The variables in the left of each cell determine the employee's payoffs, and the variables in the right determine the employer's payoffs for each combination of player's responses. ‘
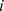
’is the cost of inspection to the employer.

For each value of the inspection cost 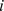, there is a unique mixed Nash equilibrium in which the probability with which the employee is shirking just corresponds to the inspection cost, 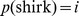, and the probability with which the employer is inspecting is 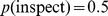. In this case, neither player can improve its expected payoff by only unilaterally changing its strategy. In fact, using 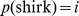, the expected payoff for the employer is always
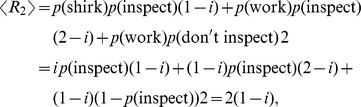
independently of 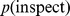. Likewise, if 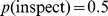, the expected payoff for the employee is always 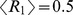, independently of 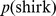.

#### pRL reproduces inspector game data and Nash equilibria

**Figure 3:**
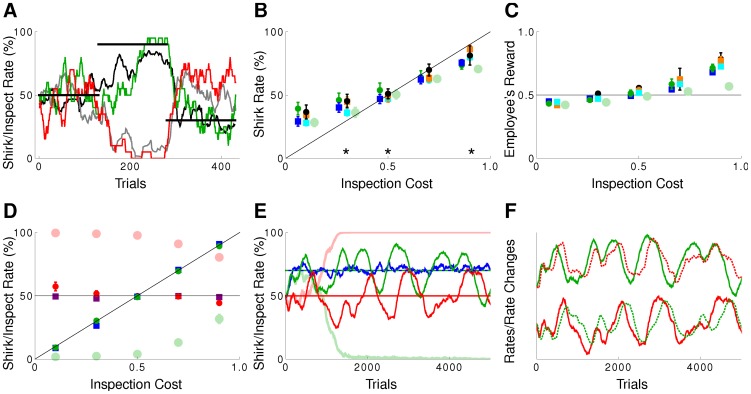
pRL but not TD-learning fits data and follows a mixed Nash equilibrium. (A) Choice behavior for pRL versus pRL (employee green, employer red) and human versus human (employee black, employer gray) [11]. The cost of inspection was stepped from 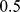 to 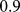 to 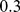, respectively, and this does also correspond to the shirk rate in Nash equilibrium (thick black lines). The inspection rate in the Nash equilibrium would always be 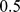. (B) Average choice behavior of pRL vs pRL (dark green circles) and TD vs TD (light green circles), pRL for the employee vs computer algorithm for the employer (blue squares), human vs human (black), human as an employee vs computer algorithm (orange) and monkey vs computer algorithm (cyan) for 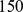 trials/block as function of the inspection cost. The solid line indicates the Nash equilibrium. (C) Reward as function of the inspection cost for 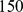 trials/block. Coloring as in (B). pRL simulations are more similar to the experimental data than the TD simulations. (D) Average choice behavior as in (B) but for 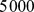 trials/block. The inspect rates for pRL vs pRL (TD vs TD) (dark (light) red circles) and pRL vs computer algorithm (purple squares) are shown too. The lines indicate the Nash equilibrium for the employee (diagonal) and the employer (horizontal). pRL behaves according to the Nash equilibrium, whereas TD does not. (E) Time course of the probability to shirk with inspection cost 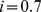 for pRL vs algorithm (blue line) and pRL vs pRL (TD vs TD) (dark (light) green line). For the latter the probability of the employer to inspect is shown too (dark (light) red line). pRL oscillates around the Nash equilibrium (drawn lines), whereas TD completely deviates from Nash. (F) Time course of the probability to shirk or inspect respectively with inspection cost 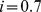 for pRL vs pRL (green respectively red, solid) as in E, but shifted up for clarity and overlaid with the negative change in the shirk rate (green dashed) and the change in the inspect rate (red dashed) to show the counteractive behavior.

We played with our neural network as employee against the algorithm presented in [11] as employer, and we also simulated two neural nets playing against each other. Fig. 3A shows a running average over the last 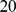 trials of the shirk and inspection rates for the neuronal net employee (green) and neuronal net employer (red), overlaid with the corresponding data for a human employee (black) playing against a human employer (grey, data from [11]). The inspection cost was held constant during three blocks of 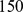 trials and stepped from 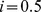 to 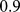 and finally to 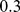. The averaged shirk rate of the neuronal net employee is in striking agreement with the one of the human employee across the whole rate of inspection costs (Fig. 3B). There is also good agreement with the experimental data for the employee's reward (Fig. 3C).

To check whether the good fit of the human data is due to the gradient property alone or whether the population boost is also necessary we considered single neurons playing against each other (by setting the number of neurons in the population to 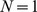, without changing the learning rule). In this case our learning rule becomes equivalent to the policy gradient rule for single escape rate neurons [29], [30]. With only a single neuron learning turns out to be too slow to match the transient behavior in the human data (Fig. 3A), even after optimizing the learning rate (data not shown). We have previously shown that the speeding up learning in a population of spiking neurons is only possible with an additional population signal modulating synaptic plasticity [22], [31]. We conclude that population learning is necessary, and that other spike-based gradient rules which do not exploit a population signal [15], [32], [33] will also be too slow.

During the 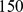 trials across an experimental block, the shirk rates (but not the inspection rates) tended towards the corresponding value of the Nash equilibrium (diagonal line in Fig. 3B), and so did the employee's reward (horizontal line in Fig. 3C), although without reaching them. In the simulation we extended the block size to check the asymptotic behavior. We found that for block sizes of 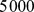 trials, the average shirk *and* the inspection rates closely reached the Nash equilibrium (match of simulation points with the two lines in Fig. 3D).

Despite the match of the average rates with the mixed Nash equilibria, the running means oscillate around the corresponding equilibria (shown in Fig. 3E for inspection cost 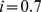), as predicted by the theory [34], [35]. In the asymmetric case, when our neuronal employee plays against the (apparently not optimal) computer algorithm, the oscillation vanish and the employee's shirk rate reaches the optimal Nash equilibrium (blue), as expected for a neuronal network endowed by synaptic modifications following the reward gradient (Supporting Text S2). When two reward maximizing networks play against each other, however, each tries to exploit any deviation of the other from his Nash equilibrium, pushing him even further away. This leads to oscillations around the Nash equilibrium where a change in strategy of one player is oppositely directed to the deviation from the Nash equilibrium of the other player. In fact, when superimposing one rate with the negative change of the other rate, a close match is observed (Fig. 3F).

As we are studying a policy gradient algorithm, one may ask how robust the described properties are in view of a possibly improper biological implementation. The eligibility trace 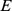 (Eq. 8 in Methods) depends on the presynaptic spike timing and contains a positive term that depends itself on the postsynaptic spike timing and a negative term depending on the postsynaptic potential. Due to the policy gradient property, the two terms are balanced and the eligibility trace is zero on average. To check for robustness we performed simulations where this balance is perturbed. The above results still qualitatively hold true if the negative term in the eligibility trace is twice as large, or even if it is neglected completely, yielding STDP with plasticity for pre-post spike pairing only. The robustness can be attributed to the factor 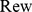 in the learning rule (Eq. 1) which averages out to 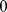 due to the subtraction of the reward prediction (since 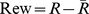, see Eq. 5 in Methods) and hence neutralizes any bias in the estimate of 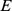 [16].

#### TD-learning is inconsistent with data and Nash

To value the match generated by our synaptic plasticity rule with experimental and theoretical data, we also trained a TD-learner on the inspector game. Yet, the parameter optimized TD-algorithm roughly reproduces only the humans average shirk rate (Fig. 3B), but less well the subjects' rewards (Fig. 3C). More strikingly, two opposing TD-learners simulated across the 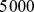 trial blocks do not behave according to the Nash equilibrium (Fig. 3D), but adopt a deterministic strategy within such a block (Fig. 3E). When simulating even longer, oscillations emerge as well, but without convergence of the long-term average to the Nash equilibrium.

There are principled reasons why TD-learning must generally fail to find an optimum solution, and in particular a mixed Nash equilibrium. First, TD-learning assumes that the underlying process is Markovian, but for multiplayer games this assumption is in general not satisfied. In fact, because the policy of the other player may change in time during learning, the optimal decision probabilities depend on past actions. Values which are assumed to be a function of the current action only, may therefore be incorrectly estimated. Second, for a mixed Nash equilibrium, TD-learning may also fail to correctly map values to decision probabilities at the steady-state after learning. This is because each policy imposes some predefined mapping from action values to decision probabilities, and this adhoc mapping may not reflect the true relationship between expected payoffs and decision probabilities defining the Nash equilibrium. For the inspector game with inspection costs 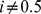, for instance, the softmax policy, which selects action 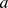 with probability 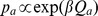, where 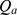 is the value of action 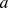 and 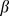 a parameter (inverse temperature) regulating the amount of stochasticity in the decision making, does never reflect the Nash probabilities 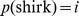 and 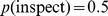, whatever the parameter 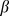 is (see Supporting Text S1).

In other learning tasks, animals and humans *transiently* match the choice probabilities with the average payoffs of the corresponding actions, 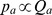 (for a discussion of probability matching see [36]–[38]). In these cases too, TD-learning with softmax will not be able to find the solutions neither, unless again all 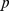
- and 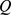-values are each the same, or alternatively, the choice policy is redefined (to 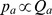, see [39]). For other examples where TD-learning fails in each of the two ways of either learning the values or inferring the choice probabilities see [7].

#### Not all covariance-rules lead to Nash equilibria

Covariance-based learning rules change the synaptic strengths 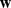 according to the covariance between reward prediction error and some measure of neuronal activity 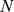, 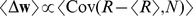, see e.g. [37], where 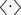 denotes expectation. pRL which follows the stochastic gradient of the expected reward, 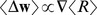, is a special instance of a covariance rule where the quantity 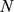 corresponds to the eligibility trace 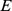 (Eq. 1). Steady-states of covariance rules satisfy Herrnstein's *matching law* [37], [40]. This law states that the number of times an action is chosen is proportional to the reward accumulated from choosing that action. Formally, 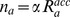, where 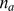 is the number of times an action 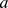 is chosen, 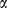 is the action-independent proportionality constant, and 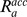 is the reward accumulated by action 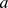 across its 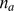 choices.

Pure strategies, where only a single action is chosen, trivially satisfy the matching property since for non-chosen actions both 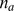 and 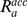 vanish. In contrast, stochastic strategies only satisfy matching in the case that the selected options provide the same average reward 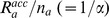. The mixed (and trivially the pure) Nash equilibrium represents a special case of matching. If in a two-player game, for instance, player 2 adopts a mixed Nash strategy then, by definition, player 1 receives the same average reward 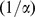 from any action (see also [38]).

**Figure 4:**
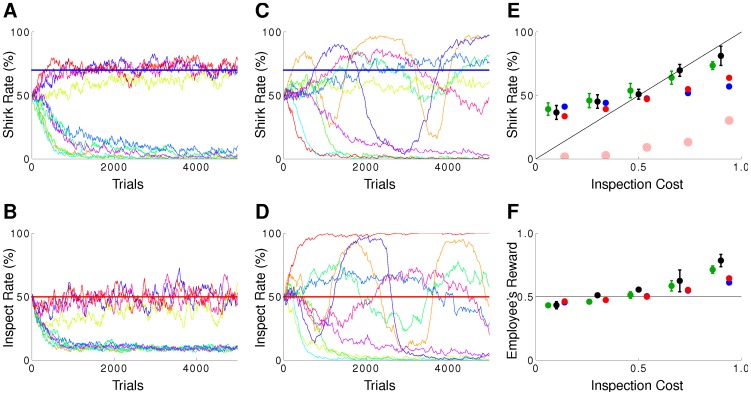
Covariance learning rules may lead to a mixed Nash equilibrium, but also to deterministic non-Nash strategies. pRL fits data better than basic reinforcement models. Time course of the probability to shirk (A,C) and inspect (B,D) with inspection cost 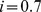 for pCOV vs algorithm (A,B) and pCOV vs pCOV (C,D). In each panel the horizontal lines depict the Nash equilibrium, and for 10 simulation runs inspection and shirk rates are shown (same color in (A,B) and (C,D), respectively, correspond to the same run). Only a small fraction of all runs converge or oscillate around the Nash equilibrium, while the other runs result in a deterministic strategy pair. The initial distribution of synaptic weights 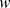 was Gauss with mean 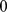 and standard deviation 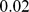. The learning rate was set to 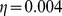, but 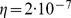 did not change the proportion of runs converging to the pure strategy. (E) Average choice behavior of pRL vs pRL (green), RE1 vs RE1 (blue), RE3 vs RE3 (red) and human vs human (black) for 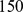 trials/block as function of the inspection cost. The light red circles show the average choice behavior for RE3 vs RE3 and 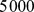 trials/block. Individual runs converged to a pure strategy, hence the shown averages over 200 runs reflect the percentage of runs converging to a pure shirk strategy. (F) Reward as function of the inspection cost for 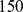 trials/block. Coloring as in (E). The solid lines indicate the Nash equilibrium.

Both the steady-state of a covariance rule and the Nash equilibrium imply matching. But a steady-state of the covariance rule does not necessarily need to be a Nash equilibrium. This can be seen by generalizing the classical covariance rule [37] to population learning, and applying this rule to the inspector game. To do so we replaced the eligibility trace 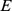 in the pRL rule (Eq. 1) by the deviation of the neuronal response 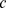 from its mean, 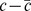. The emerging population covariance (pCOV) learning rule,
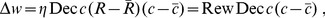
(2)with positive learning rate 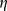 and estimates 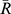 (see Eq. 5 in Methods) and 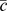 for 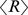 and 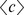 respectively, does not follow the reward gradient and does not reliably converge to the unique mixed Nash equilibrium of the inspector game (Fig. 4). To keep simulation time short we did not use spiking neurons but merely binary neurons that produce output 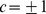 with probability 
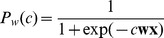
 for a given binary input pattern 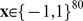. This yields an expected neural response 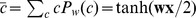. Notice that 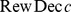 can be considered as the personalized reward for the specific neuron in consideration: if the sign of 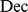 and 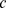 coincide, the population reward signal 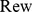 elicited in response to the population decision can be taken as a personal reward signal for that neuron; otherwise the neuron's personal reward has reversed sign of 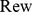. Personalizing this way the neuronal rewards within the population solves the spatial credit-assignment problem and boosts learning [22]. Fig. 4 (A–D) shows the results for the pCOV rule applied to the inspector game, once with only the employee playing according to pCOV against an employer playing according to the algorithm presented in [11], and once for pCOV versus pCOV. Only in a fraction of the simulated runs is the mixed Nash equilibrium reached, while in the other runs, a deterministic (non-Nash) strategy pair emerges.

As check for robustness we performed further simulations showing that these negative results hold true also for other (non-gradient) covariance rules. We considered the version where the mean 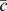 is not calculated analytically but determined as a running average (as done for 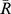), and where the neuronal activity in the covariance rule is equal to the binary output, 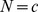, without mean subtraction (yielding the simple update rule 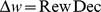). Moreover, considering only a single neuron and taking its response 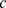 as the behavioral decision did not qualitatively change the results, demonstrating that the failure is not due to the population framework (data not shown).

In [41] the author further elaborates on the relationship of covariance based rules to the Replicator dynamics. The latter is described by 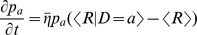, where 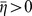 is the effective learning rate and 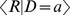 is the average reward for choosing action 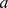. The effective learning rate 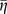 depends on the details of the decision making network and is given in Eq.(14) of [41]. If synaptic changes are driven by the covariance of reward and neural activity, then according to the average velocity approximation, learning behavior is described by the differential equation above, but the effective learning rate 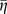 is not guaranteed to be positive. Indeed, for the binary neurons one gets 
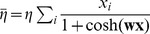
, which can be negative due to negative components 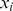. Hence, the convergence statements in [41] do not apply to our decision making network, and there is no contradiction with the finding that the covariance rule fails to reproduce the mixed Nash equilibrium. Nevertheless, in the special case of a 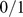 coding of the inputs 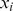 such that the effective learning rate becomes positive, and the specific covariance rule 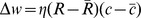 with a single postsynaptic neuron (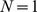), we can also fit the human data (for 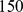 learning trials) and obtain the oscillations around the Nash equilibrium (for 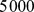 trials).

Whereas we do not consider the neural mechanisms supporting the readout of the decision, such a mechanism has been studied in the context of matching [42]. There, the probability for decision making is well described by a logistic function, which is also our choice for 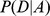. For an increasing amount of stochasticity in the decision making they report an increasing deviation from matching towards more uniform choice probabilities. For choice probabilities larger than 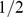 this leads to lower choice probabilities than predicted by the matching law, a phenomenon called undermatching. We also varied the amount of stochasticity by changing the slope of 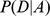 as a function of 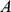. We find that increasing and decreasing the slope by a factor of two still robustly leads to matching for the considered inspection costs, but when decreasing it by a factor of ten we also observe undermatching. Due to the stochasticity in the decision making the range of choice probabilities our network can represent is limited. With increasing the amount of stochasticity the range becomes smaller and extreme probabilities close to 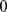 or 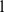 predicted by the matching law cannot be represented. Instead, choice probabilities lie closer to uniform randomness, i.e. undermatching occurs.

#### pRL fits data and Nash better than basic reinforcement models

The Replicator equation is widely used in evolutionary game theory and provides a good phenomenological description of choice behavior in many repeated-choice experiments. A different phenomenological description has been suggested in [17]. Starting from Luce's basic reinforcement learning (RE1, see also [43]), the authors adapt this rule to take account of a generalization and recency effect (RE3, Methods). They show that both the basic and extended reinforcement learning reproduces the behavior of humans in many different games. However, we find that these rules poorly match human behavior for the inspector game. In fact, for 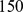 trials/block both models of Erev and Roth fit the experimental data significantly worse than pRL (Fig. 4 E and F). After 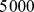 trials RE3 converged to a pure (non-Nash) strategy, and for RE1 the inspection rate diverged away from the Nash equilibrium of 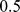. This non-optimal equilibrium performance is consistent with the fact that RE1 and RE3 are not gradient procedures (see Supporting Text S3). Whether pRL fits the behavioral data of humans also better in the other games Erev and Roth considered remains to be tested. In any case, the models have to cope with the fact that humans show a variety of behaviors in two-player matrix games, although in many settings they eventually play according to Nash (for a discussion see [44]).

## Discussion

We considered a population of spiking neurons which represent an adaptive agent in a dynamic environment including other adaptive agents. The agent's adaptation was implemented as population reinforcement learning algorithm (pRL) which was previously shown to perform stochastic gradient ascent in the reward for partially observable Markov decision processes (POMDPs) [7]. Here we showed with blackjack and the inspector game that pRL can also cope with a dynamic multi-agent environment and that the performance is comparable to human data in both these games. In fact, when two neuronal populations play against each other, they learn to behave according to the optimal (but unstable) Nash equilibrium. By definition, no further increase in an agent's expected payoff is possible in the Nash equilibrium by only changing its own strategy while the environment remains stationary. In these steady-state conditions – where the opponent's strategy is assumed to be stationary – pRL is proven to maximize the expected reward (Supporting Text S2). The simulations show that the equilibrium is indeed reached by two pRL agents playing against each other, with a pure (deterministic) Nash equilibrium in blackjack and a mixed (stochastic) Nash equilibrium in the inspector game. As predicted by the theory [34], [35], the strategies oscillated around the mixed Nash equilibrium when both players used the same gradient algorithm based on the others stationarity assumption, i.e. when one network played against another both using pRL (with a small learning rate). Averaging over long enough time windows, i.e. long compared to the oscillation period, yields the Nash equilibrium values. However, when implementing only the employee by a gradient pRL network and the employer by a non-gradient computer algorithm [11], the two players do not play exactly equally well. In this case no oscillations occurred and both converged to and stayed at the optimal Nash equilibrium.

For mathematical clarity we presented the spike-based pRL for an episodic learning scenario. But a biologically plausible implementation of a fully online scheme is also possible: to avoid an explicit separation of stimuli in time, the rectangular window function used to temporally integrate the eligibility trace (Eq. 8 in Methods) can be replaced by an exponentially decaying window function to get a low-pass filtered eligibility trace, and concentrations of neuromodulators can be used to encode feedback about the population decision and the global reward signal (e.g. acetylcholine or dopamine) [22]. We considered reward delivery immediately after stimulus presentation, but reward could also be substantially delayed when considering a further eligibility trace incorporating the population decision [7]. Moreover, since learning in general speeds up with population size (up to 1-shot learning for stimulus-response associations [31]) we expect that the convergence for pRL towards the Nash equilibrium can be much faster than in our example where parameters were fit to reproduce human data.

The mixed Nash equilibrium represents a special case of Herrnstein's matching law [40], according to which the number of times an action is chosen is proportional to the reward accumulated from choosing that action. This is true both for the pure and mixed Nash optimum. In the special case that the current reward only depends on the current action, but not on past actions, reward maximization always implies matching. (In fact, if one action would yield a higher (average) payoff per choice, then this action must be chosen with probability 1 to maximize expected reward, and matching (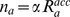) is trivially satisfied (since for the non-chosen action 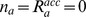). If both actions yield the same payoff 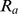 per choice (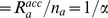), then matching is again trivially satisfied.) In turn, a reward-based learning rule which only empirically maximizes reward in this case leads to only an approximated matching [42]. Choice probabilities which maximize the expected reward are trivially also fixed points of any learning rule defined by the covariance between reward and neuronal activity. (In fact, at the reward maximum there is no change in neuronal activity which, in average, would lead to an increase (and in the opposite direction to a decrease) of the expected reward, and hence the covariance between activity and reward must vanish.) The other direction, again, is not true: a covariance-based rule does not necessarily lead to reward maximization or a Nash equilibrium [37], [38]. Indeed, our simulations of the inspector game with the canonical covariance-based plasticity rules show that these rules do not necessarily lead to the mixed Nash equilibrium, but instead can result in deterministic (non-Nash) strategies. Similarly, basic reinforcement rules studied in the context of economics and human decision making [17] are neither compatible with the mixed Nash equilibrium for the inspector game.

The performance of spike-based pRL is also superior to TD-learning [2] which is often discussed in the neuro-economical context [1]. With the parameter values for which TD-learners came closest to human data (although without matching them as closely as pRL), the mixed Nash equilibrium in the inspector game was not reached within the long learning times. Instead, TD-learner first adopted a deterministic strategy, transiently switched their behavior, and swapped back to the same deterministic strategy. We attributed this mismatch to a general failing of TD-learning in correctly mapping action values to choice probabilities in probabilistic decision making tasks. TD-learning with the softmax choice policy, in particular, fails when matching of choice probabilities with average payoff is required [40].

Different generalizations have been considered to approach the shortcomings of algorithms in socio-economic games. TD-learning has been extended to not only assign values to its own decisions, but to pairs of own and opponent decisions. This enables the learning of minimax strategies where reward is maximized for the worst of the opponents actions [45]. While for zero-sum games minimax may realize a mixed Nash equilibrium, it results in a deterministic strategy in the inspector game: minimizing the maximal loss implies for the employee to always work (to prevent being caught while shirking), and for the employer to always inspect (to prevent undetected shirking). Another approach is to separately learn its own and the opponents action values and then calculate the Nash equilibrium [46], but such explicit calculations do not seem to be the typical human behavior in socio-economic interactions. Instead, it is tempting to consider pRL with long eligibility traces which, as it performs policy gradient in POMDPs [7], should find cooperative strategies with, on average, higher than Nash payoffs for all agents. For the inspector game such a co-operative strategy is that the employer should let the employee sporadically shirk (say with probability 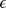) without inspection, but with the common agreement that shirking will not prevail (leading to average payoffs 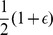 and 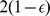 for the employee and employer, respectively).

Although under the specific experimental conditions of the inspector game humans did not show cooperation, they often do so in other game-theoretic paradigms, as e.g. in the prisoner's dilemma, and hence deviate from the Nash equilibrium (for a review see [47]). It remains a challenge for future modeling work to capture such cooperative behavior. Likely, this will involve modeling the prediction of other player's reactions in response to ones own actions, as considered in the theory of mind [48] and as being a hallmark of successful socio-economic behavior.

Given the difficulties of modeling genuine social behavior, and the difficulties humans effectively have in stacked reflexive reasoning, the assumption of the opponent's stationarity considered here appears as a reasonable approximation for decision making even in complex situations. In view of its success in matching behavioral and theoretical data we may ask how far human decision making is in fact determined by cognitive reasoning, or whether decisions should rather be attributed to automated neuronal processes steered e.g. by pRL (which can also encompass input from a cognitive module as it is suggested for the production rules in the ACT-R theory, [18]). In fact, daily experience tells us that decisions are often more appropriate when we listen to our gut feeling, while we tend to merely add justifications post-hoc. Or put in Schopenhauer's words, “that in spite of all his resolutions and reflections he does not change his conduct” [49].

## Methods

### Model details

Focusing on one neuron we denote by 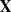 its input, which is a spike pattern made up of 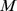 spike trains, and by 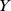 its output spike train. The membrane potential can be written as
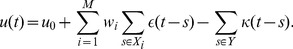
(3)The postsynaptic kernel 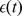 and the reset kernel 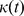 vanish for 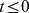. For 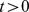 they are given by
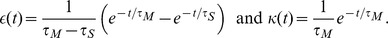
For the resting potential we use 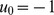 (arbitrary units). Further, 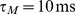 is used for the membrane time constant and 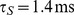 for the synaptic time constant. Action potential generation is controlled by an instantaneous firing rate 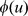 which increases with the membrane potential. So, at each point 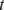 in time, the neuron fires with probability 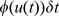 where 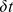 represents an infinitesimal time window (we use 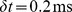 in the simulations). Our firing rate function is
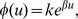
with 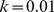 and 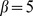 (parameter values taken from [29], see also [19]).

We consider a population of 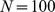 neurons and an input layer of size 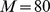 for each player that is represented by a neural net. We assume that each population neuron synapses onto a site in the input layer with probability of 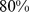, leading to many shared input spike trains between the neurons. The population response is read out by the decision making unit based on a spike/no-spike code. We introduce the coding function 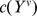, with 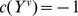, if neuron 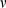 does not spike, otherwise 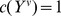. The population activity 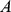 being read out by the decision making unit is:
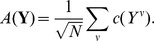
Note that such a formal summation could be implemented in terms of a neuronal integrator (forming a ‘line attractor’) which continuously integrates excitatory and inhibitory input and keeps the neuronal activity at a constant level in the absence of input [50]. Using this activity readout, the behavioral decision 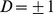 is made probabilistically, with likelihood 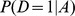 given by the logistic function
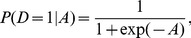
(4)and 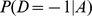 being the counter probability. The normalization of the activity 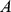 with 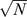 ensures that 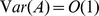, thus being of same order as the noise in the decision readout.

We now describe the terms, modulating synaptic plasticity in Eq. (1). The reward feedback 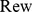 encodes the reward prediction error, as observed in experiments [20],
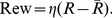
(5)Here 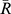 is a running mean estimate of the expected reward, 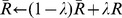, where we set 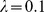. The parameter 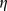 is the positive learning rate which, for notational convenience, we absorb into the reward signal. In all pRL simulations we used the value 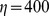. Both values 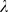 and 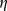 (rounded) were chosen to minimize the Mean Squared Error (MSE) between the average model and human data (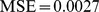 for the shirk rate and 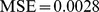 for the employee's reward in the inspector game). All other parameter values were taken from [7].

The decision feedback 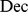 is given by
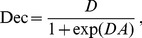
(6)which is the derivative of 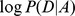, see Eq. (4), with respect to 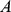; so decision feedback measures how sensitive the decision is to changes in activity.

As shown in [29], the probability density, 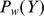, that a neuron actually produces the output spike train 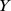 in response to the stimulus 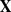 during a decision period lasting from 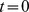 to 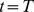 satisfies:
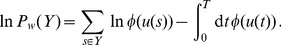
(7)The derivative of 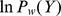 with respect to the strength of synapse 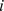 is known as characteristic eligibility in reinforcement learning [51]. For our choice of the firing rate function one obtains for the last term in (1)
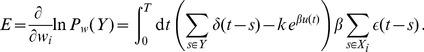
(8)


In all the simulations initial values for the synaptic strength were picked from a Gaussian distribution with mean zero and standard deviation equal to 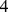, independently for each afferent and each neuron. In the Supporting Text S2 we show that the plasticity rule (1) composed of the factors (5, 6, 8) and the decision 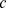 follows the stochastic gradient of the expected reward.

### TD-learning

For TD-learning we used the SARSA control algorithm [2] which estimates the values of state-action pairs 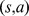. At each point in time, the value estimates 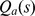 are updated according to
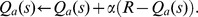
Here 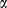 is similar to a learning rate and has values between 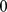 and 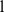. 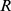 is the reward immediately obtained after performing action 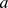. In the case of blackjack it is defined as zero if the game is not over and the player chooses to draw another card, otherwise it is determined by the payoffs of the considered game. When in state 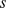, the next action 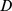 is chosen using softmax, i.e. according to the probability 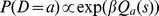. In all simulations we used the rounded values 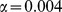 and 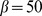 as they minimized the MSE between averaged model and human data (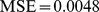 for the shirk rate and 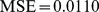 for the employee's reward in the inspector game). Note that in both TD-learning and pRL we adapted the same number of free parameters (TD: 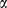 and 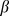; pRL: 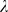 and 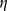), making it possible to directly compare the quality of the fit.

### Basic reinforcement models

In both Roth-Erev models [17] the probabilistic choice rule is parametrized using propensities 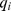. The probability 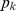 that a specific player (who's index is omitted) plays his 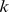
th pure strategy is 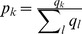.

#### RE1 model

The propensity of the chosen action 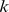 is incremented by the received reward 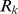, 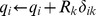, where 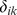 denotes the Kronecker delta. The initial propensities are set to 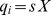, where 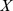 is the average reward for that player under uniformly distributed 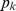
's, and the strength parameter 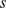 is the one parameter that is optimized.

#### RE3 model

In addition to the strength parameter 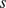, the generalization and forgetting parameters 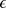 and 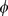 are introduced. The propensities are updated according to
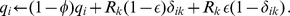
The parameters were chosen to minimize the mean squared error (MSE) between the average model and human data (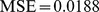 (RE1) and 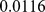 (RE3) for the shirk rate and 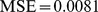 (RE1) and 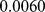 (RE3) for the employee's reward in the inspector game).

### Blackjack details

In blackjack we assume an infinite number of card decks. Independently of the history, the drawing probability therefore remains constant, with a probability to draw a card with value 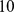 being 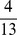, and the probability to draw any other value from 2 to 11 being 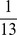. For a strategy determined by the stopping value 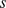 we calculated analytically the probability distribution of hand values 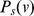 after drawing the last card. The drawing process is iterated for those hand values that are smaller than 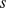 until there is only probability mass on hand values greater than or equal to 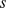. Because the lowest card value on the desk remains always 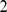, drawing 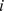 times in a row yields a lowest possible hand value of 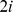. Hence up to 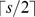 cards are drawn in order to obtain a hand value 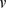 greater or equal to 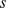. Let us denote the value of the 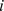
th card by 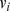 and its probability distribution by 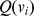,
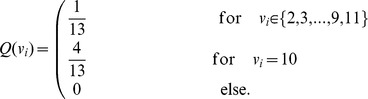
To obtain the probability distribution 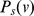 we sum up the probabilities of all possible combinations to draw 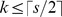 cards that yield hand value 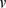, with the condition that the sum of the first 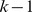 drawn cards is smaller than 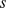, such that a 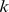
th card is actually drawn under the stopping strategy.
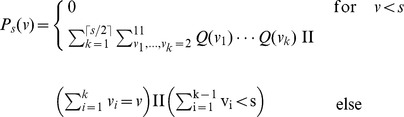
where II is the indicator function which is one if its argument is true and zero else. The product of the 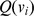 is the joint probability that the first card has value 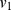, the second 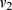 and so on. The first indicator function ensures that all 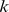 drawn cards sum up to 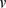, the second that 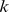 cards are drawn, i.e. the sum of the first 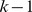 cards has to be smaller than the stopping value 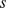, because otherwise no further card would be drawn. For instance in the case of 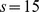 and 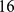 one obtains the distributions in Table 3.

**Table 3:** Probability distribution of hand values 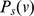 after drawing the last card.

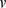	<15	15	16	17	18	19	20	21	>21
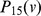	0	0.1206	0.1247	0.1194	0.1138	0.1078	0.1546	0.0944	0.1648
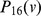	0	0	0.1247	0.1287	0.1231	0.1170	0.1638	0.1036	0.2390

Denoting the hand value of the gambler by 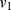 and that of the croupier by 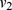 the payoff of the bank is
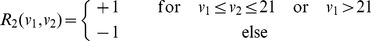
Averaging of 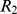 with respect to the joint distribution 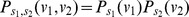 yields the entry in the average payoff matrix Table 1 for the strategy pair (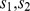). For instance for (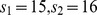), 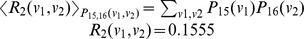.

We defined the drawing probabilities in Fig. 2 for a hand value at a certain game number 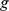 as the frequency with which another card has been drawn upon the last 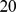 presentations prior to 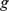 of the corresponding stimulus. The evolution of the average reward 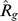 in time in Fig. 2C are the low pass filtered reward sequences, 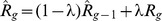 where 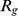 is the reward in the 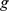-th game and 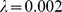 was used. The initial value 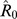 was calculated assuming a random 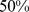 choice behavior prior to learning.

The initial weights mimicking the prior strategy of instructed humans were obtained by training our network to make a decision with a certain probability. This is possible by adapting pRL to perform regression (as will be published elsewhere).

### Inspector game details

The evolution of the rates in time in Fig. 3E are the low pass filtered decision sequences, e.g. 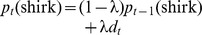 where 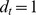 if the employee shirks in trial 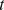, otherwise 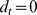. We used a value of 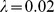 and assumed again an initial random 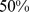 choice behavior. The rate change in Fig. 3F was determine by binning the obtained time course of the rate into bins of width 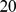, calculating the mean of each bin, and the differences between succeeding bins. The result was further low pass filtered once more with an exponential running mean (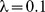) to reduce the noise.

## Supporting Information

Text S1 We present further results for temporal-difference learning and elaborate on its failure to learn mixed Nash equilibria.
(PDF)

Text S2 We show how the plasticity rule presented in the main text is based on a gradient ascent procedure maximizing the average reward.
(PDF)

Text S3 We demonstrate that the heuristic rules of Erev and Roth [17] are no gradient procedures.
(PDF)
