# Supplementary material for: Spike-based Decision Learning of Nash Equilibria in Two-Player Games
Source: PLoS Comput Biol. 2012 Sep 27;8(9):e1002691. doi: 10.1371/journal.pcbi.1002691 (PMC3459907; doi:10.1371/journal.pcbi.1002691)
Supplement: Text S1 — We present further results for temporal-difference learning and elaborate on its failure to learn mixed Nash equilibria. (PDF) [file pcbi.1002691.s001.pdf]

# Spike-based decision learning of Nash equilibria in two-player games.
# Text S1: Further results for temporal-difference learning

Johannes Friedrich[1], Walter Senn[1,*]
**1 Department of Physiology and Center for Cognition, Learning and Memory, University of Bern, Bühlplatz 5, CH-3012 Bern, Switzerland**
∗ **E-mail: senn@pyl.unibe.ch**

In this Supplementary Material we present further results for TD-learning and elaborate on its failure to learn mixed Nash equilibria.

## TD successfully learns a pure Nash equilibrium

TD-learning of blackjack yields similar results as spike-based population reinforcement learning (pRL). In Fig. S1 results for both algorithms are shown. TD learns the optimal deterministic decisions, even in the non-Markovian case of two TD-learners playing against each other (Fig. S1A).

## TD fails to learn a mixed Nash equilibrium

The resulting asymptotic choice behavior of TD-learning in the inspector game depends on the inverse temperature in the softmax action selection. The Nash equilibrium is never reached, though for one TD-learner playing against the algorithm one can find an inverse temperature where the behavior is at least close to Nash (Fig. S2A). For two TD-learners (Fig. S2B) we calculated the asymptotic behavior by demanding that the average value update equals zero, i.e. the Q-values equal the expected payoffs. Using softmax action selection and consulting the payoff table Tab.2 this yields the following system of

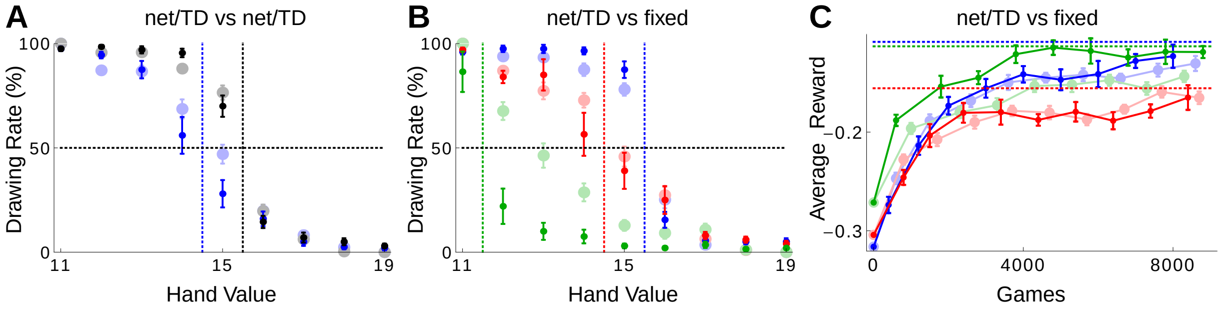


**Figure S1. Playing blackjack yields similar results for pRL and TD.** (A) Average strategy (±SEM) after 10 000 games where the gambler (blue) is a neural net (small dark circles) or TD-learner (large light circles) as well as the croupier (black). The vertical dotted lines left of $s_1 = 15$ and $s_2 = 16$ show the separation line of drawing/not drawing another card for the optimal Nash strategy pair. (B) Average strategy (±SEM) after 10 000 games for a neural net (small dark circles) or TD-learner (large light circles) as gambler playing against a croupier that follows a given strategy $s_2 = 15$ (blue), 16 (red) or 17 (green). The colored dotted lines left of $s_1 = 12, 15, 16$ show the separation line of drawing/not drawing another card for the optimal strategy given that the croupier stops drawing at $s_2 = 17, 16, 15$ (from left to right). (C) Average reward (±SEM) of the gambler for the scenario described in (B). The colored dotted lines show the maximal reachable average reward.

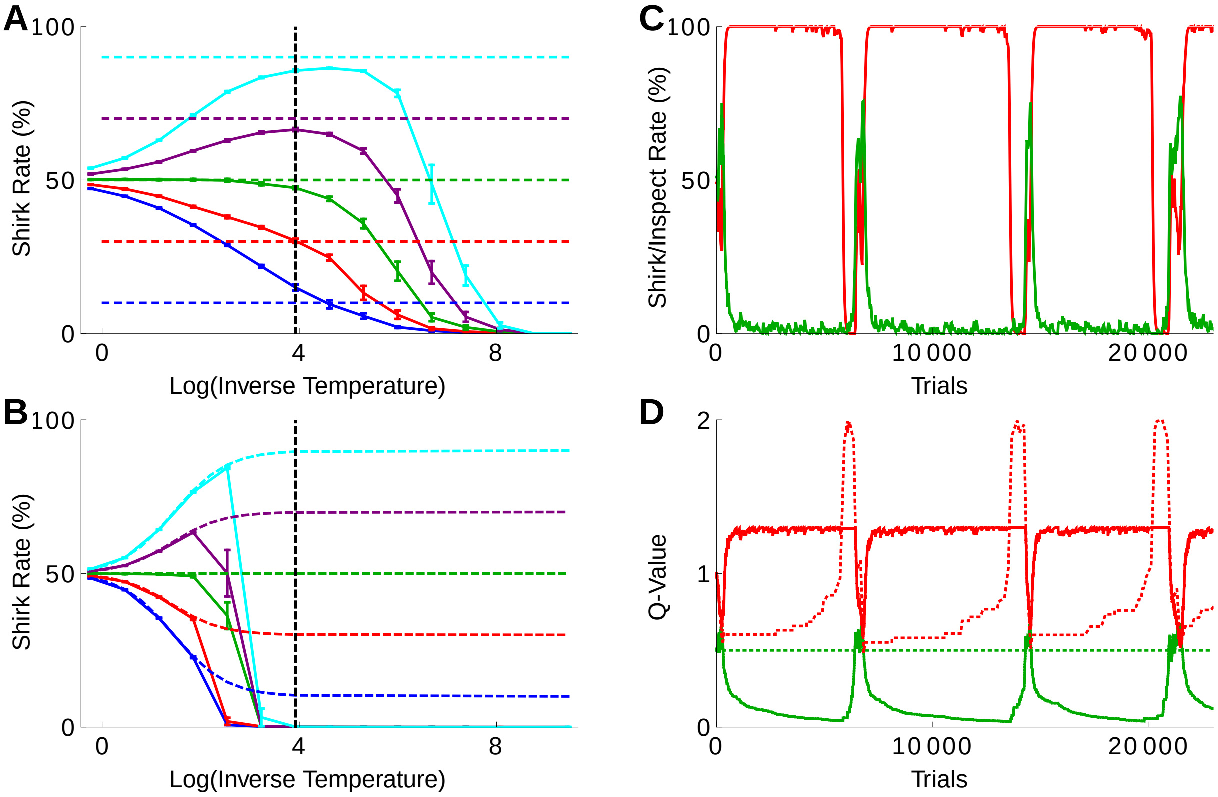


**Figure S2. TD-learning in the inspector game strongly depends on the inverse temperature and shows oscillations.** (A) Average choice behavior for TD vs computer algorithm over 5 000 trials as function of the inverse temperature $\beta$ for inspection cost $i = 0.1$ (blue), 0.3 (red), 0.5 (green), 0.7 (purple) and 0.9 (cyan). The colored dashed lines indicate the Nash equilibrium, the black dashed line the value $\beta = 50$ chosen in Fig. 3 of the main text to best fit the experiments. (B) Average choice behavior for TD vs TD. The colored dashed lines show the solution of the equation system obtained when the average TD-update equals zero. (C) Single run shirk rate (green) and inspect rate (red) as function of time for TD vs TD. (D) Q-value of shirking (solid green), working (dashed green), inspecting (solid red) and not inspecting (dashed red) as function of time for the same single run as in (C). In (A) and (B) the same value $\alpha = 0.004$ as in the main text was used, whereas in (C) and (D) the parameters were set to $\alpha = 0.2$ and $\beta = 10$ in order to demonstrate the oscillatory behavior of TD-learning. With the values used in the main text the period of the oscillations would exceed reasonable simulation times.

equations:

$$p_s = \frac{e^{\beta Q_s}}{e^{\beta Q_s} + e^{\beta Q_w}} = \frac{1}{1 + e^{\beta(Q_w - Q_s)}} \quad \text{(S1)}$$

$$p_i = \frac{e^{\beta Q_i}}{e^{\beta Q_i} + e^{\beta Q_n}} = \frac{1}{1 + e^{\beta(Q_i - Q_n)}} \quad \text{(S2)}$$

$$Q_s = 1 - p_i \quad \text{(S3)}$$

$$Q_w = 0.5 \quad \text{(S4)}$$

$$Q_i = (2 - i)(1 - p_s) + (1 - i)p_s \quad \text{(S5)}$$

$$Q_n = 2(1 - p_s) \quad \text{(S6)}$$

where we introduced the notations $p_s = p(\text{shirk})$, $p_i = p(\text{inspect})$, $Q_s = Q(\text{shirk})$, $Q_w = Q(\text{work})$, $Q_i = Q(\text{inspect})$ and $Q_n = Q(\text{don't inspect})$. Given the inspection costs $i$ and some inverse temperature $\beta$, one can plug in the Q-values (Eqs. (S3)–(S6)) into Eqs. (S1) and (S2) and solve for $p_s$ and $p_i$. It turns out that, unless $i = 0.5$, the Nash equilibrium $p_s = i$ and $p_i = 0.5$ is never a solution of this system for any $\beta$. Hence, TD learning can never find the Nash equilibrium.

We numerically solved the above system for different $i$ and $\beta$ and plotted $p_s$ as a function of $\beta$ for different values of $i$ (Fig. S2B). For increasing values of $\beta$ the shirk probability $p_s$ comes closer to the Nash value $p_s = i$. But for large $\beta$'s slightly imprecise estimates of the Q-values will push the shirk and inspection probabilities to either 0 or 1, as can be seen from the very right of Eqs. (S1) and (S2). In numerical simulations this is expressed by the oscillations found in the action rates and the Q-values (Fig. S2C,D). There are long phases were the employee nearly always works and the employer inspects. Given that the employee shirks rarely, choosing to not inspect would actually yield a higher payoff for the employer, but the value of this action is still low and only updated in exploratory steps. Finally it surpasses the value of inspection and there is a phase were the employer does not inspect, which is realized after some exploratory steps by the employee who starts shirking. This leads to a drastic change in the employer's payoff and the employer's Q-values drop quickly to a low level. The employer inspects more forcing the employee to work. When the employee works again the payoff of the employer increases which he attributes to his inspection. The Q-value for inspection increases rapidly while the option to not inspect is rarely taken and its Q-value remains low, thus the cycle repeats. The period of the cycle is determined by how often exploratory steps are taken and due to the softmax action selection grows exponentially with the inverse temperature.
