# Supplementary material for: Spike-based Decision Learning of Nash Equilibria in Two-Player Games
Source: PLoS Comput Biol. 2012 Sep 27;8(9):e1002691. doi: 10.1371/journal.pcbi.1002691 (PMC3459907; doi:10.1371/journal.pcbi.1002691)
Supplement: Text S2 — We show how the plasticity rule presented in the main text is based on a gradient ascent procedure maximizing the average reward. (PDF) [file pcbi.1002691.s002.pdf]

Spike-based decision learning of Nash equilibria in two-player games.
Text S2: Relating the plasticity rule to a gradient ascent procedure

Johannes Friedrich[1], Walter Senn[1,*]
1 Department of Physiology and Center for Cognition, Learning and Memory, University of Bern, Bühlplatz 5, CH-3012 Bern, Switzerland
∗ E-mail: senn@pyl.unibe.ch

Here we show how the plasticity rule presented in the main text is based on a gradient ascent procedure. First, a formula is derived for the gradient of the probability of taking a behavioral decision with respect to synaptic strength of the population neurons. Finally, we show that the plasticity rule presented in the main text performs stochastic gradient ascent in the expected reward. The derivation is analog to [7], but without delayed reward.

Gradient for the behavioral decision

Let $\mathbf{X}$ be the spike pattern presented to the population neurons and $\mathbf{W}$ the matrix of their synaptic strength. The probability $P_{\mathbf{W}}(D)$ of responding with decision D to the stimulus X is

$$P_{\mathbf{W}}(D) = \int \mathrm{d}\mathbf{Y}\, P(D|A(\mathbf{Y})) \prod_{\nu=1}^{N} P_{\mathbf{W}^\nu}(Y^\nu)\,, \tag{S7}$$

where we suppressed the conditioning on X. To lighten the notation further, we focus on calculating the gradient of $P_{\mathbf{W}}(D|X)$ only with respect to the strength of one of the synapses (the expressions for the other synapses being entirely analogous). Let w denote the strength of the first synapse of the first population neuron and let $Y = Y^1$ the postsynaptic spike train produced by this neuron. To isolate the contribution of the first neuron we decompose the activity $A(\mathbf{Y})$ as

$$A(\mathbf{Y}) = \tfrac{1}{\sqrt{N}} c(Y) + A^{\backslash}(Y^2, \ldots, Y^N) \quad \text{with } A^{\backslash} = \frac{1}{\sqrt{N}} \sum_{\nu=2}^{N} c(Y^\nu)\,.$$

Plugging this into (S7) we can calculate the derivative of $P_{\mathbf{W}}(D)$ with respect to the single weight w performed in the Supplementary Materials of [7]. Changing the matrix index of $P_{\mathbf{W}}(D)$ to w we obtain

$$\tfrac{\partial}{\partial w} P_w(D) = \int \mathrm{d}Y \mathrm{d}A^{\backslash} P_w(D, A^{\backslash}, Y)\ \tfrac{1}{\sqrt{N}} \left(\tfrac{\partial}{\partial A} \ln P(D|A)\right) c(Y) \tfrac{\partial}{\partial w} \ln P_w(Y)\,. \tag{S8}$$

Gradient of the expected reward

Since we consider immediate reward application without delay (unlike in [7]), reward does only depend on the decisions D_1 and D_2 of both opposing agents in the current trial. We assume that the first agent is a population of neurons, whereas the decision making process of the second agent remains unspecified,

leaving the formalism general. The derivative of the first agent's expected reward $\langle R_1 \rangle$ in that trial is

$$\begin{aligned}
\frac{\partial}{\partial w}\langle R_1\rangle &= \frac{\partial}{\partial w}\sum_{D_1,D_2} P_w(D_1,D_2)R(D_1,D_2) \\
&= \sum_{D_1,D_2} P(D_2|D_1)R(D_1,D_2)\frac{\partial}{\partial w}P_w(D_1) \\
&= \sum_{D_1,D_2} P(D_2|D_1)\left(R(D_1,D_2)-\bar{R}\right)\frac{\partial}{\partial w}P_w(D_1)\,. \qquad \text{(S9)}
\end{aligned}$$

In the last line we subtracted a term equaling zero for a reward baseline $\bar{R}$ that is conditionally independent of the current decisions D_1 and D_2, given the weights and the stimulus [51]. The choice of an adaptive estimate $\bar{R}$ of upcoming reinforcement based on past experience is known as reinforcement comparison [2]. The common approach we follow to compute $\bar{R}$ is to use the exponential averaging scheme. Formally, we assume that the probability distribution of the second agent's decisions conditioned on D_1, $P(D_2|D_1)$, is stationary. Plugging (S8) into (S9) yields

$$\begin{aligned}
\frac{\partial}{\partial w}\langle R\rangle = \sum_{D_1,D_2}\int &\mathrm{d}Y\mathrm{d}A^{\setminus}\, P_{w,t}(D_2,D_1,A^{\setminus},Y) \\
&\times\left(R(D_1,D_2)-\bar{R}\right)\frac{1}{\sqrt{N}}\left(\frac{\partial}{\partial A}\ln P(D_1|A)\right)c(Y)\frac{\partial}{\partial w}\ln P_w(Y) \qquad \text{(S10)}
\end{aligned}$$

The first line is just the averaging operator. We can now compare the terms in the second line to the weight update (1),

$$\Delta w = \mathrm{Rew}\,\mathrm{Dec}\,c\,E\,, \qquad \text{(S11)}$$

proposed in the main text. The first term corresponds to the reward signal $\mathrm{Rew} = \eta(R-\bar{R})$ given by Eq.(5). The derivative in the second term yields for the logistic function $P(D|A) = 1/(1+\exp(-DA))$ in Eq.(4) the decision feedback $\mathrm{Dec} = D/(1+\exp(DA))$ given by Eq.(6). The last term has already been introduced as eligibility trace E in Eq.(8). If we choose a small learning rate, the average over the decisions D_1, D_2, the postsynaptic spike train Y and the activity of the other neurons $A^{\setminus}$ can be replaced by a time average obtained by sampling these quantities. The corresponding online learning rule (1,S11) therefore results from dropping the averaging. While the transition from batch to online learning requires a small learning rate for the neuronal population even in a stationary environment, here further the learning rate of the second agent has to be small too, i.e. $P(D_2|D_1)$ changes slowly in time. On the other side the learning rate should not be too small in order to be able to react to changes in the dynamic environment.
