# Supplementary material for: Spike-based Decision Learning of Nash Equilibria in Two-Player Games
Source: PLoS Comput Biol. 2012 Sep 27;8(9):e1002691. doi: 10.1371/journal.pcbi.1002691 (PMC3459907; doi:10.1371/journal.pcbi.1002691)
Supplement: Text S3 — We demonstrate that the heuristic rules of Erev and Roth [17] are no gradient procedures. (PDF) [file pcbi.1002691.s003.pdf]

Spike-based decision learning of Nash equilibria in two-player games.
Text S3: The Roth-Erev models are no gradient procedures

Johannes Friedrich[1], Walter Senn[1,*]
1 Department of Physiology and Center for Cognition, Learning and Memory, University of Bern, Bühlplatz 5, CH-3012 Bern, Switzerland
∗ E-mail: senn@pyl.unibe.ch

In this Supplementary Material we show that the Roth-Erev models [17] do not update the propensities in the gradient direction of the reward. For convenience we restate the update of the propensities for the 3-parameter model of Erev an Roth (ER3),

$$q_i \leftarrow (1-\phi)q_i + R_k(1-\epsilon)\delta_{ik} + R_k\,\epsilon\,(1-\delta_{ik})\,. \tag{S12}$$

Remember that the choice probabilities are set to $p_i = q_i/\sum_l q_l$. The one parameter model (ER1) is just a special case thereof with $\epsilon = \phi = 0$.

In a stochastic gradient procedure the average propensity change is proportional to the gradient of the expected reward. The latter is, suppressing the index n of the player, $\frac{\partial}{\partial q_i}\langle R\rangle = \sum_k p_k R_k \frac{\partial}{\partial q_i}\ln p_k$ where the last term evaluates to $\frac{\partial}{\partial q_i}\ln p_k = \frac{\delta_{ik}}{q_k} - \frac{1}{\sum_l q_l}$. The ensuing update rule is

$$q_i \leftarrow q_i + \eta\, R_k \left(\frac{\delta_{ik}}{q_k} - \frac{1}{\sum_l q_l}\right)\,, \tag{S13}$$

where the positive parameter η is the learning rate. The update differs from the update of RE3 (S12) for any choice of parameters.

To conclude already that RE3 is not a policy gradient procedure would be one step too fast. There are many different estimates for the reward gradient, $R_k(\frac{\delta_{ik}}{q_k} - \frac{1}{\sum_l q_l})$ is just one of them and maybe RE3 uses another one. We have to consider whether the *average* updates are equal. For the gradient procedure we obtain from averaging across the choice options $k = 1, 2$,

$$\langle \Delta q_i^{grad}\rangle = \eta\frac{\partial}{\partial q_i}\langle R\rangle = \eta\left(\frac{p_i R_i}{q_i} - \frac{\langle R\rangle}{\sum_l q_l}\right) = \frac{\eta}{\sum_l q_l}\left(R_i(1-p_i) - p_j R_j\right)\,, \tag{S14}$$

where i is one and j the other option. In contrast, for RE3 we obtain

$$\langle \Delta q_i^{RE}\rangle = -\phi q_i + p_i R_i(1-\epsilon) + p_j R_j \epsilon \tag{S15}$$

The average propensity update $\langle \Delta q_i^{RE}\rangle$ (S15) is never equal to $\langle \Delta q_i^{grad}\rangle$ (S14) for any parameter setting, hence the rule does not perform (stochastic) gradient ascent in the expected reward.
